# Emotional and Cognitive Perspectives in Physical Activity and Sport: An Introduction

**DOI:** 10.3390/bs15060801

**Published:** 2025-06-11

**Authors:** Yair Galily, Gershon Tenenbaum

**Affiliations:** 1 Sport, Media and Society (SMS) Research Lab, Sammy Ofer School of Communications, Reichman University (IDC) Herzliya, Herzliya 46150, Israel; gtenenbaum@fsu.edu; 2 Baruch Ivcher School of Psychology, Reichman University (IDC) Herzliya, Herzliya 46150, Israel

## 1. Introduction

In the rapidly evolving world of physical activity and sport science, researchers and practitioners alike are increasingly recognizing that the path to optimal performance and well-being is paved not just with physical conditioning and technical skill but also with emotional regulation and cognitive acuity (Lepers et al., 2025; Mercader-Rubio et al., 2023; Quan et al., 2025; Terzioğlu & Çakir-Çelebi, 2025). No longer can peak performance be understood solely through metrics such as speed, strength, or endurance. Instead, it must be examined through a more holistic lens—one that integrates the athlete’s inner emotional landscape, mental resilience, attentional control, and capacity for adaptive decision-making under pressure (Samuel et al., 2024). As athletes and exercisers navigate increasingly complex sporting environments, where physical demands are compounded by psychological intensity and social expectations, the importance of emotional and cognitive processes becomes not only evident but central.

This Special Issue, “*Emotional and Cognitive Perspectives on Physical Activity and Sport*”, reflects this growing awareness, providing a multidisciplinary platform for research exploring the intricate interplay between how individuals move, feel, and think. It brings together a body of work that transcends disciplinary silos, drawing from and contributing to psychology, neuroscience, education, philosophy, and kinesiology. The eight contributions collected in this volume offer compelling insights with diverse cultural contexts, methodological frameworks, and theoretical foundations. From large-scale empirical studies and innovative interventions to meta-analyses and conceptual commentaries, these papers illuminate the nuanced roles played by emotion and cognition across a variety of sporting and physical activity domains.

Collectively, they reflect the complexity and richness of a field that sits at the crossroads of multiple disciplines—a field that is as concerned with ethical questions and philosophical underpinnings as it is with performance statistics and physiological measures. This Special Issue reaffirms that emotional and cognitive dimensions are not mere embellishments to physical activity but foundational components that shape how sport is experienced, taught, studied, and performed.

Central to this issue is the shared conviction that emotions are not peripheral to sport—they are constitutive of it. They influence motivation, shape interpersonal dynamics, modulate stress responses, and can either elevate or undermine performance. Likewise, cognition is not merely a backdrop to performance but an active process that guides, adapts, and sometimes transforms it. Decision-making, attentional focus, learning, and self-regulation are all deeply embedded in the sporting act, influencing not only outcomes but also personal growth, identity formation, and psychological resilience.

## 2. Objectives

Our objective as Guest Editors was to cultivate a space where these interdependencies could be explored with empirical depth, theoretical innovation, and practical relevance. We aimed to highlight research that not only diagnosed problems or documented trends but also suggested solutions, sparked debate, and opened up new avenues for inquiry. The result is a Special Issue that challenges traditional boundaries, invites interdisciplinary collaboration, and affirms the centrality of emotional and cognitive processes in understanding the full human dimension of sport and physical activity.

A foundational theme running throughout this Special Issue is the role of mindfulness and its relationship with stress regulation. In their article, “*Does Self-Reported Trait Mindfulness Contribute to Reducing Perceived Stress in Women Who Practice Yoga and Are Physically Active?*”, Cavour-Więcławek and Rogowska (2024) investigated how regular engagement with yoga and physical activity was correlated with increased mindfulness traits and reduced stress among women. Their cross-sectional study of 201 participants revealed that those who practiced yoga for at least 150 min per week reported significantly lower levels of perceived stress and higher mindfulness than their less active counterparts. These findings underscore the potential of contemplative physical practices not only in physical health but as key instruments in achieving emotional balance and resilience. Cavour-Więcławek and Rogowska’s work compellingly suggests that the act of moving the body in a mindful, intentional way can reshape internal emotional landscapes.

This intersection of physical activity and emotional development continues to be explored in the article by Wang et al. (2024), “*The Relationship between Participation in Extracurricular Arts and Sports Activities and Adolescents’ Social and Emotional Skills*.” Drawing on rich data from a student survey in Suzhou, China, the authors employed Ordinary Least Squares and coarsened exact matching techniques to examine how extracurricular involvement shaped social–emotional competencies. Their findings show that adolescents who engage in both sports and arts programs exhibit greater emotional intelligence, empathy, and cooperative behaviors. These effects are not only statistically significant but also synergistic, meaning that the combination of creative and athletic engagement offers more profound developmental benefits than either domain alone. Wang, Li, and Yao’s research situates sport and art as dual vehicles for cultivating emotional maturity and social competence—skills that are foundational to both academic and life success.

Further exploring the educational and developmental potential of sport, Wang and Qian (2024) present the study “*The Mediating and Moderating Role of Social–Emotional Skills in the Relationship between Sports Participation and Test Anxiety*”. Leveraging a massive sample of over 60,000 adolescents from the OECD Social and Emotional Skills Survey, their study reveals that social–emotional skills not only mediate but also moderate the link between sports participation and test anxiety. Interestingly, the most significant buffering effects are observed among adolescents with lower or moderate levels of these skills, indicating that sport may serve as a compensatory context for students who are otherwise psychologically vulnerable. Wang and Qian’s work brings granularity to the idea that not all students benefit from physical activity equally and that interventions should be tailored to the psychological profiles of participants.

Whereas the above studies emphasize developmental stages and educational contexts, for their contribution, *Empowering Movement: Enhancing Young Adults’ Physical Activity through Self-Determination Theory and Acceptance and Commitment Therapy-Based Intervention*”, Lev-Arey et al. (2024) applied a therapeutic lens to young adult behavior. Their program, titled “Running Minds”, combines principles from Self-Determination Theory (SDT) and Acceptance and Commitment Therapy (ACT) to increase physical activity engagement among inactive young adults. Through an eight-session intervention focused on aligning values with goals and fostering emotional acceptance, participants experienced improved motivation, increased physical activity levels, and enhanced emotional self-regulation. Lev-Arey, Gutman, and Levental’s innovative approach illustrates the power of integrating cognitive–behavioral frameworks into motivational theory to create holistic, psychologically informed physical activity programs.

The intersection of emotional influence and strategic cognition was further investigated in a unique context by Tenenbaum et al. (2023) for their article “*Coaches’ Mind Games: Harnessing Technical Fouls for Psychological Momentum in Basketball*”. Through qualitative interviews with Israeli first-division basketball coaches, the authors unpack how technical fouls—often perceived as rash emotional outbursts—are frequently used as deliberate tools to shift game momentum, influence referees, and energize players. The study reveals a calculated manipulation of emotional energy, in which coaches weigh potential benefits against risks to orchestrate psychological turning points in competition. Tenenbaum, Vigodsky, and Lev’s findings bring to light a little-discussed aspect of coaching psychology and highlight the strategic role of emotional expression in high-pressure environments.

While the contributions above explore the impact of emotional and cognitive processes on current behavior, the article by Yang et al. (2024), “*Relationship between Athletes’ Big Five Model of Personality and Athletic Performance: Meta-Analysis*”, examines stable personality traits as predictors of athletic outcomes. Conducting a systematic review and meta-analysis of 18 studies using the PRISMA framework, the authors identified conscientiousness and extraversion as the personality traits most consistently associated with high athletic performance. While other traits showed less clear effects, the meta-analysis underscored the importance of integrating personality assessments into talent development and coaching. Yang and colleagues offer a valuable perspective on how long-term, dispositional attributes interact with short-term performance metrics and suggest pathways for personalized coaching based on psychological profiling.

Complementing this long-view approach is the culturally specific and temporally sensitive work of Bae et al. (2024), “*The Model of Goal-Directed Behavior in Sports Participation: A Meta-Analysis Comparing Pre- and Post-COVID-19 Eras in the Republic of Korea*.” By comparing 18 studies conducted before and after the pandemic, the authors assess how the relationships between motivational variables and sports consumption behavior have shifted in response to societal change. Their findings reveal significant moderating effects caused by the pandemic, especially regarding subjective norms and perceived behavioral control, offering insights into how public health crises recalibrate collective and individual attitudes toward sport. Bae, Chiu, and Nam’s work demonstrates the necessity of flexible theoretical models that can accommodate the fluid nature of psychological drivers in times of disruption.

This Special Issue concludes with a philosophically charged commentary by Galily (2024), “*From Sport Psychology to Action Philosophy: Immanuel Kant and the Case of Video Assistant Referees*”. In a thought-provoking exploration, Galily examines the ethical implications of technological intervention in sport, using Kantian ethics to analyze the growing reliance on Video Assistant Referees (VARs) in soccer. He argues that while VARs may enhance fairness, they also challenge the spirit of the game by undermining spontaneity and human judgment. The commentary invites readers to consider not just whether technology works but whether it should be used in the ways in which it currently is. Galily’s philosophical analysis challenges the field to move beyond empirical data and toward normative questioning about the values that we wish to preserve in sport.

Together, the following eight articles form a coherent yet multifaceted tapestry, each contributing to a deeper, more integrated understanding of the emotional and cognitive forces that shape physical activity and sport:

1. Bae, J. S., Chiu, W., & Nam, S. B. (2024). The model of goal-directed behavior in sports participation: A meta-analysis comparing pre-and post-COVID-19 eras in the Republic of Korea. Behavioral Sciences, 14(7), 556.
2. Cavour-Więcławek, N., & Rogowska, A. M. (2024). Does self-reported trait mindfulness contribute to reducing perceived stress in women who practice yoga and are physically active? Behavioral Sciences, 14(9), 772.
3. Galily, Y. (2024). From sport psychology to action philosophy: Immanuel kant and the case of video assistant referees. Behavioral Sciences, 14(4), 291.
4. Lev-Arey, D., Gutman, T., & Levental, O. (2024). Empowering movement: Enhancing young adults’ physical activity through self-determination theory and acceptance and commitment therapy-based intervention. Behavioral Sciences, 14(2), 130.
5. Tenenbaum, G., Vigodsky, A., & Lev, A. (2023). Coaches’ mind games: Harnessing technical fouls for psychological momentum in basketball. Behavioral Sciences, 13(11), 904.
6. Wang, W., Li, W., & Yao, J. (2024). The Relationship between Participation in Extracurricular Arts and Sports Activities and Adolescents’ Social and Emotional Skills: An Empirical Analysis Based on the OECD Social and Emotional Skills Survey. Behavioral Sciences, 14(7), 541.
7. Wang, K., & Qian, J. (2024). The Mediating and Moderating Role of Social-Emotional Skills in the Relationship between Sports Participation and Test Anxiety. Behavioral Sciences, 14(6), 512.
8. Yang, J. H., Yang, H. J., Choi, C., & Bum, C. H. (2024). Relationship between athletes’ big five model of personality and athletic performance: Meta-analysis. Behavioral Sciences, 14(1), 71.

All the articles in this Special Issue remind us that beneath the physical gestures of sport—every pass, jump, shot, or run—lie complex psychological processes, deep emotional currents, and socially constructed meanings. Whether in youth development, elite coaching, behavioral intervention, or philosophical inquiry, these forces are at once powerful and nuanced.

Several cross-cutting themes emerge from the collective insights of this Special Issue. Firstly, the synergy between emotional well-being and physical activity is not accidental but systematic. Multiple articles demonstrate that when physical engagement is paired with reflective, intentional, or social–emotional learning, its benefits are magnified. Secondly, there is a growing consensus that psychological constructs—be they traits such as conscientiousness or states such as mindfulness—can and should inform intervention strategies, coaching practices, and policy development. Finally, this Special Issue underscores the importance of cultural and contextual specificity, reminding us that emotional and cognitive processes are shaped not only by biology and behavior but also by history, society, and ethics.

## 3. Conclusions

The field of physical activity and sport has experienced a significant transformation in recent years, marked by an increasing acknowledgment of the emotional and cognitive dimensions that underpin performance, motivation, learning, and well-being (Beenen et al., 2025; Doorley et al., 2022; Hoffmann et al., 2022; Jekauc et al., 2021; Jin et al., 2025). Contemporary research has moved beyond traditional performance indicators such as strength, speed, and endurance, and has begun to embrace a more holistic understanding of the athlete—one that recognizes the critical influence of emotional regulation, cognitive flexibility, attentional control, and psychological resilience. Despite these advances, a persistent gap remains in terms of fully integrating these psychological components into the broader landscape of sport science, particularly in ways that are empirically grounded, culturally sensitive, and applicable across diverse populations and levels of participation.

This Special Issue, *Emotional and Cognitive Perspectives in Physical Activity and Sport*, responds directly to that gap by presenting a curated collection of research and commentary works that traverse disciplinary, methodological, and cultural boundaries. These contributions explore a wide range of topics, including the impact of mindfulness on stress reduction, the role of emotional intelligence in youth development, cognitive–behavioral strategies for increasing physical activity among young adults, and even the philosophical implications of technological interventions in sport. By addressing both stable traits and dynamic states and by combining large-scale quantitative studies with nuanced qualitative insights, this Special Issue offers a multidimensional portrayal of the athlete as a thinking, feeling, and socially embedded individual. The research presented here not only enriches our theoretical understanding but also lays the groundwork for evidence-based practices in coaching, physical education, mental skill training, and policy-making.

As the field moves forward, future research should focus on several key directions. Longitudinal studies are needed to capture the evolving interplay between emotional and cognitive factors and physical engagement over time. Intervention-based research should be prioritized to assess the real-world effectiveness of strategies designed to enhance psychological well-being through sport. Additionally, greater attention should be paid to the cultural, socioeconomic, and contextual variables that mediate these processes, ensuring that insights are relevant and inclusive across different communities and settings. Interdisciplinary collaboration—linking psychology, neuroscience, education, ethics, and sport science—will be essential in developing robust, adaptable models that reflect the complexity of human behavior in athletic and physical activity environments.

Ultimately, this Special Issue affirms that in order to truly understand and enhance human movement, we must engage with the full spectrum of human experience—emotional, cognitive, social, and philosophical. It is our hope that the research presented here will inspire continued inquiry, provoke thoughtful debate, and pave the way for future innovations that honor the deeply interconnected nature of mind and body in sport.

As editors, we extend our deepest thanks to the authors, the reviewers, and the editorial team at *Behavioral Sciences* for their contributions. This Special Issue would not be possible without their intellectual rigor, methodological integrity, and creative vision. We hope that these articles will serve as touchstones for future research, inspire interdisciplinary collaboration, and contribute to a richer understanding of the deeply human experience of sport.
